# Unraveling a 146 Years Old Taxonomic Puzzle: Validation of Malabar Snakehead, Species-Status and Its Relevance for Channid Systematics and Evolution

**DOI:** 10.1371/journal.pone.0021272

**Published:** 2011-06-24

**Authors:** Allen Benziger, Siby Philip, Rajeev Raghavan, Palakkaparambil Hamsa Anvar Ali, Mithun Sukumaran, Josin C. Tharian, Neelesh Dahanukar, Fibin Baby, Reynold Peter, Karunakaran Rema Devi, Kizhakke Veetil Radhakrishnan, Mohamed AbdulKather Haniffa, Ralf Britz, Agostinho Antunes

**Affiliations:** 1 Department of Zoology, Fatima Mata National College, Kollam, Kerala, India; 2 Centre for Aquaculture Research and Extension, St. Xavier's College, Palayamkottai, Tamil Nadu, India; 3 CIMAR/CIIMAR, Centro Interdisciplinar de Investigação Marinha e Ambiental, Universidade do Porto, Porto, Portugal; 4 Departamento de Biologia, Faculdade de Ciências, Universidade do Porto, Porto, Portugal; 5 Conservation Research Group, St. Albert's College, Kochi, Kerala, India; 6 Durrell Institute of Conservation and Ecology, School of Anthropology and Conservation, University of Kent, Canterbury, Kent, United Kingdom; 7 Concert for Ecology and Applied Technology, Kochi, Kerala, India; 8 Institute of Tropical Aquaculture, Universiti Malaysia Terengganu, Kuala Terengganu, Malaysia; 9 Department of Zoology and Environmental Science, St. Johns College, Anchal, Kerala, India; 10 Indian Institute of Science Education and Research, Pune, Maharashtra, India; 11 Marine Biotechnology Division, Central Marine Fisheries Research Institute, Kochi, Kerala, India; 12 Zoological Survey of India, Southern Regional Centre, Chennai, Tamil Nadu, India; 13 Ichthyology Laboratory, School of Life Sciences, South China Normal University, Guangzhou, Guangdong, People's Republic of China; 14 Department of Zoology, The Natural History Museum, London, United Kingdom; Biodiversity Insitute of Ontario - University of Guelph, Canada

## Abstract

**Background:**

The Malabar snakehead *Channa diplogramma* is one of the most enigmatic and least understood species within the family Channidae, which comprise one of the most important groups of freshwater food fish in tropical Asia. Since its description from peninsular India in 1865, it has remained a taxonomic puzzle with many researchers questioning its validity, based on its striking similarity with the South East Asian *C. micropeltes*. In this study, we assessed the identity of the Malabar snakehead, *C. diplogramma*, using morphological and molecular genetic analyses, and also evaluated its phylogenetic relationships and evolutionary biogeography.

**Methodology/Principal Findings:**

The morphometric and meristic analysis provided conclusive evidence to separate *C. diplogramma* and *C. micropeltes* as two distinct species. Number of caudal fin rays, lateral line scales, scales below lateral line; total vertebrae, pre-anal length and body depth were the most prominent characters that can be used to differentiate both the species. *Channa diplogramma* also shows several ontogenic color phases during its life history, which is shared with *C. micropeltes*. Finally, the genetic distance between both species for the partial mitochondrial 16S rRNA and COI sequences is also well above the intra-specific genetic distances of any other channid species compared in this study.

**Conclusions/Significance:**

The current distribution of *C. diplogramma* and *C. micropeltes* is best explained by vicariance. The significant variation in the key taxonomic characters and the results of the molecular marker analysis points towards an allopatric speciation event or vicariant divergence from a common ancestor, which molecular data suggests to have occurred as early as 21.76 million years ago. The resurrection of *C. diplogramma* from the synonymy of *C. micropeltes* has hence been confirmed 146 years after its initial description and 134 years after it was synonymised, establishing it is an endemic species of peninsular India and prioritizing its conservation value.

## Introduction

Freshwater fishes comprise one of the most diverse groups of vertebrates with an estimated 13,000 species worldwide, and many more waiting to be described in the tropics, especially in countries where exploratory surveys are still incomplete such as China and India [1]. In the Southern Indian state of Kerala, where this study was based, 10–20% of the fishes in any basin of reasonable size are thought to be undescribed [2]. This slow rate of progress in fish species assessments and identification is largely due to the lack of funding and trained taxonomists in these regions, all of which contribute to the ‘taxonomic impediment’ [3].

Snakeheads of the genus *Channa* comprise one of the most important groups of freshwater food fish in tropical Asia [4], with a wide natural distribution extending across the continent from Iran in the West, to China in the East, and parts of Siberia in the Far East [5]. They are one of the most common staple food fish in Thailand, Cambodia, Vietnam and other South East Asian countries where they are extensively cultured [4], [6]. Apart from their importance as a food fish, snakeheads are also consumed as a therapeutic for wound healing as well as reducing post-operative pain and discomfort [7], and collected for the international aquarium pet trade [8].

The taxonomy of the genus *Channa* remains incompletely known, as a comprehensive revision of the family has not been performed, and more new species continue to be described. Therefore, an uncertainty still exists regarding the total number of species within this genus. Of the 87 nominal species and 4 subspecies that have been described, many are now considered synonyms of recognized species, and there are about 20 names that cannot be associated with any valid taxa [9]. It has also been suggested that as many as five species *viz*, *C. gachua*, *C. marulius*, *C. micropeltes*, *C. punctata*, and *C. striata* may in fact represent “species complexes” [9], [10]–[14]. A recent phylogenetic study has also indicated the likelihood of the existence of more undescribed species of channids in South East Asia [14].

The Malabar snakehead, *Channa diplogramma* is one of the most enigmatic and least known of all channids. Sir Francis Day [15] described *Ophiocephalus diplogramma* in 1865 based on one juvenile specimen (42 mm in length) collected near the mouth of the Cochin River in the port city of Cochin (Southwestern India), and called it Malabar snakehead (Holotype at the Natural History Museum, London; BMNH 1865.7.17.24). The color pattern of this juvenile matched with that of juveniles of another species of snakehead, *O. micropeltes* originally described by Cuvier and Valenciennes [16] from Java, Indonesia. This possibly led Francis Day to synonymise *C. diplogramma* with *C. micropeltes* in 1878 [17]. The close similarity, rarity of adult specimens in museum collections, and the fact that no taxonomist has studied this snakehead since its description, resulted in the acceptance of the synonymy by subsequent taxonomists [9], [18]–[20]. However, recent researchers [14], [21] suggested that *C. diplogramma* is distinct from *C. micropeltes* and should be considered as a valid species.

**Figure 1:**
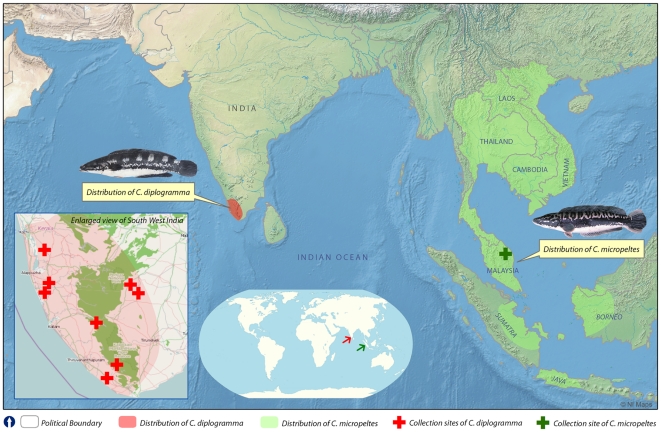
Map showing the distribution range of *Channa diplogramma* and *Channa micropeltes*.

In peninsular India, from where *C. diplogramma* was described (Fig. 1), this species has long been identified and documented as *C. micropeltes* [19], [20], [22]–[26]. But there have also been opinions that the species recorded as *C. micropeltes* from India is actually a distinct species [27], and that it is *C. diplogramma* [28]. There are also others who have suggested that both *C. micropeltes* and *C. diplogramma* occur in India [29], while another school of thought was that *C. micropeltes* was introduced, prior to mid 1800′s, to South India from South East Asia since Cochin was a major port with trading activity for many centuries [9].

The primary aim of this paper was to resolve the taxonomic ambiguity, and discuss the identity as well as systematic position of the Malabar snakehead, *C. diplogramma*, using morphological and molecular genetic (mitochondrial 16S rRNA and COI gene) information, in addition to making an attempt to understand its phylogenetic relationships and evolutionary biogeography. Both morphological and genetic analyses support *C. diplogramma* as a distinct and valid species endemic to peninsular India and reveal its importance for conservation.

## Methods

### Biometry

Measurements and counts followed those in standard literature on channid taxonomy [30]–[31]. Rays were counted with a binocular microscope and vertebral counts were taken from radiographs. The following abbreviations are used in the text: SL, standard length and TL, total length. Institutional abbreviations: BMNH – Natural History Museum, London, United Kingdom; RMNH - Rijksmuseum van Natuurlijke Histoire RMNH/Naturalis, Leiden, The Netherlands; NHM – Natural History Museum, Vienna, Austria; UMT – Universiti Malaysia Terengganu, Kuala Terengganu, Malaysia; CRG- Conservation Research Group, Department of Aquaculture, St. Albert's College, Kochi, India.

**Figure 2:**
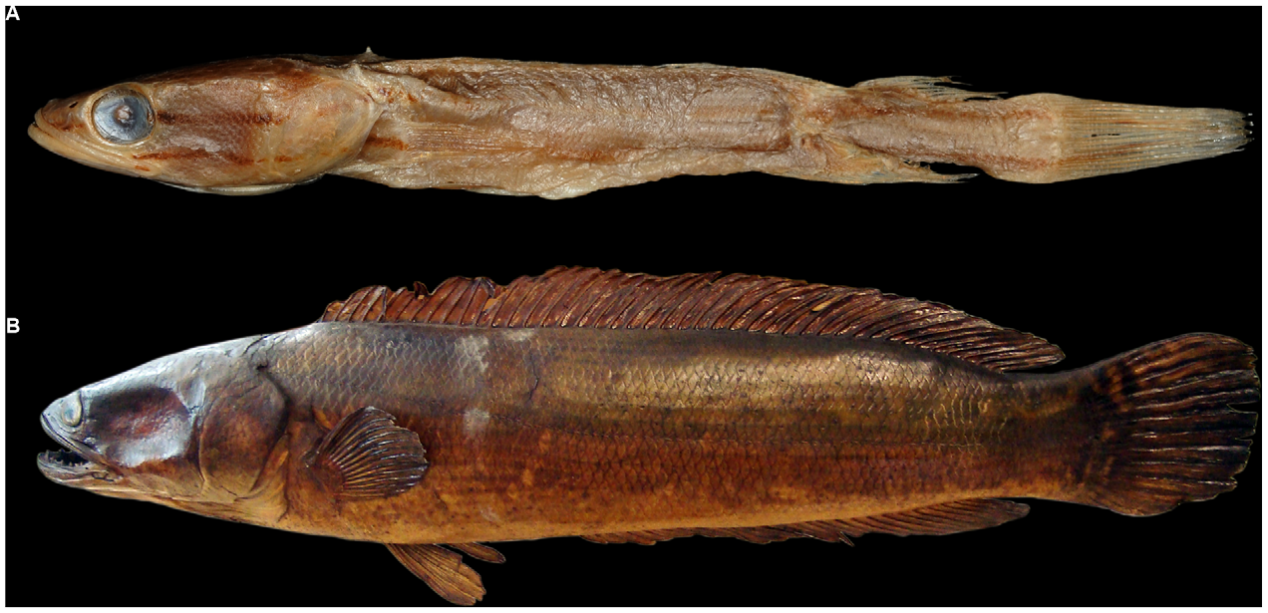
Types specimen examined in the study. A) *Channa diplogramma* (BMNH 1865.7.17.24) B) *C. micropeltes* (RMNH D2318).

Ten individuals of the Malabar Snakehead were collected from the Rivers Meenachil (9.65° N & 76.59° E) and Pamba (9.36° N & 76.53° E) in Kerala, India and five individuals of *C. micropeltes* collected from Tasik Kenyir Lake (4.96° N & 102.70° E) in Terengganu State, Malaysia. At the first stage, the morphometric and meristic characters of these fresh specimens were matched and confirmed with those of the type specimens of both species (RMNH D2318, BMNH 1865.7.17.24) (see Table S1 and Fig. 2 for details and measurements of the type specimen). Since the types of *C. micropeltes* were dry (stuffed) specimens, with missing fin rays and dry/damaged scales, we could not do a complete morphometric assessment. We therefore used only the measurements of fresh specimens to do the statistical analyses. The measurements were compared using a two-tailed unpaired t test. For some of the meristic characters where one species did not show any variation, we performed one-sample *t* test with the character value of the species showing no variation as the hypothetical mean. Principle Component Analysis (PCA) was performed on the morphometric characters (measured as % TL) and meristic characters using a correlation matrix between the variables to nullify the size and unit effect. The PCA was performed in Statistica 10® and the PCA biplot was plotted using the freeware Biplot 1.1 [32].

Voucher specimens of *C. diplogramma* examined in our study are currently deposited at the museum of CRG, Department of Aquaculture, St. Albert's College, Kochi, India (CRG-CHDIP-20-CRG-CHDIP- 29), while those of *C. micropeltes* at the Museum of the Institute of Tropical Aquaculture, Universiti Malaysia Terengganu, Kuala Terengganu, Malaysia (UMTCM1 to UMTCM5).

### DNA extraction, amplification, sequencing and analysis

The total genomic DNA of two individuals each from six of the eight *Channa* species found in India (*C. aurantimaculata, C. bleheri, C. gachua, C. marulius, C. punctata, C. striata*), six individuals of *C. diplogramma* (River Meenachil, India; 9.65° N and 76.59° E) and one individual of *C. micropeltes* (Tasik Kenyar, Malaysia; 4.96° N & 102.70° E) were isolated using a modified salting out protocol [33]. Details of the specimens used for the molecular analysis, voucher numbers and museum details are given in Table S2. Approximately 600 base pair (bp) fragments of the mitochondrial (mtDNA) 16S rRNA and Cytochrome c Oxidase subunit 1 (COI) genes were amplified from each of these eight species of *Channa* using 1µl of the DNA extract as a template, and using the following primers; L2510 (5′CGC CTG TTT ATC AAA AAC AT 3′) and H3080 (5′ CCG GTC TGA ACT CAG ATC ACG T 3′) for the 16S rRNA gene [34], FishR2-(5′ TCA ACC AAC CAC AAA GAC ATT GGC AC 3′), FishR1- (5′ TAG ACT TCT GGG TGG CCA AAG AAT CA 3′), FishF2-(5′ TCG ACT AAT CAT AAA GAT ATC GGC AC 3′), and FishF1- (5′ ACT TCA GGG TGA CCG AAG AAT CAG AA 3′) for the COI gene [35]. The amplifications were performed in 25µl reactions containing 1x assay buffer (100 mM Tris, 500 mM KCl, 0.1% gelatin, pH 9.0) with 1.5 mM MgCl2, 10 p moles/µL of primer mix, 10 mMdNTPs), 1.5 U Taq DNA polymerase and 20 ng of template DNA. To evaluate the reliability of the DNA amplification, a negative control was set up by omitting the template DNA from the reaction mixture. The reaction mixture was initially denatured at 95°C for 5 minutes followed by 29 cycles [denaturation at 94°C for 45 seconds, annealing at 50°C (for 16S rRNA) or 54°C (for COI) for 30 seconds and 72°C for 45 seconds]. Reaction was then subjected to a final extension at 72°C for 5 minutes. The PCR products were then cleaned up and subsequently sent for sequencing.

The DNA sequences were edited using BIOEDIT [36] and aligned using MUSCLE [37]. Relationships among the mtDNA haplotypes were assessed using neighbor-joining (NJ) and maximum-likelihood (ML) algorithms in SEAVIEW [38] and PHYML [39], respectively. Before carrying out the Maximum likelihood analysis the best fit nucleotide substitution model was determined using MrAIC [40]. *Notopterus notopterus* was used as an out-group species for all the analyses. A concatenated dataset of both COI and 16S rRNA sequences was prepared to produce a final phylogenetic tree.

### Genetic Distance Calculation

Using the best fit nucleotide substitution model the gamma shape parameter was calculated. The estimated value of shape parameter for the discrete Gamma Distribution was 0.2424 for 16S rRNA and 0.2238 for COI. Substitution pattern and rates were estimated under the General Time Reversible model + gamma (GTR+G) with five rate categories. Analyzes were conducted using the Maximum Composite Likelihood method [41] in MEGA5 [42]. The rate variation among sites was modeled using the previously calculated gamma shape parameter. The differences in the composition bias among sequences were considered in the evolutionary comparisons [43]. All ambiguous positions were removed for each sequence pair.

### Phylogenetic tree calibration and divergence time estimation

We used four different tree calibration methods, the Non Parametric Rate Smoothening (NPRS) and its variant NPRS-LOG [44], the Global Rate Minimum Deformation Method (GRMD) and the Local Rate Minimum Deformation Method (LMRD) [45]. The NPRS cost functions have the disadvantage of being asymmetric, but the latter two methods are perfectly symmetric. We implemented 10000 replicates to each method, which produced a two-dimensional array of data replicates, which was then calibrated by rate smoothing. Finally, the mean and confidence limit of rates and divergence times were computed from their observed distribution among the replicate sample.

A calibration file was prepared (expression written in the special purpose Treefinder's language) to implement the calibration constraints in Treefinder [45]. We used two different constraints on the channid phylogenetic tree. The node separating the genus *Parachanna* from *Channa* was constrained to 50 million years ago (MYA), which corresponds to the earliest channid fossil records from the early Eocene [46]. The fossils, Kuldana and Chorgali formations of *Anchichanna kuldanensis*, and another fossil, *Eochanna chorlakkiensis*, from Chorlakki, both located in the North West Frontier Province of Pakistan, are from deposits believed to be of similar age [47]. The alternative constraint applied of 110–84 MYA corresponds to the emergence of the genus *Channa* [48].

## Results

### Taxonomy

Taxonomic status of *Channa diplogramma* (Day 1865):

Family: Channidae

Genus: Ophiocephalus (Bloch 1793)

Genus: Channa, Scopoli 1777

Ophiocephalus diplogramma Day 1865 [15]

Ophiocephalus diplogramme Day 1865 [49]

Ophiocephalus micropeltes non Cuvier 1831 [17]

*Channa micropeltes* (non Cuvier 1831) [9], [18]–[20], [50]

*Channa diplogramma* (Day 1865) [14], [21]

### Comparative material

*Channa micropeltes:* RMNH D2318, 605 mm SL, Java (Syntype); RMNH D1131, 210 mm SL, Java & D1132 250 mm SL, Java (both possible syntypes); four specimens collected from Tasik Kenyar Lake, Terengganu, Malaysia, deposited at the Institute of Tropical Aquaculture, Universiti Malaysia Terengganu, Kuala Terrengganu, Malaysia (UMT CM1 to UMT CM5).

*Channa diplogramma:* BMNH 1865.7.17.24, 81.6 mm SL, Malabar, India (Holotype: Unique); NMW 73835, 352 mm SL, Canara, India; NMW 73838, 230 mm SL, Mangalore, India; NMW 84220, 380 mm SL, Canara, India; Six specimens collected from Meenachil River, Kerala, India and four specimens collected from Pamba River, Kerala, India deposited at the Museum of the Conservation Research Group, St. Albert's College, Kochi, India (CRG-CHDIP 20 to CRG-CHDIP 29).

### Diagnosis

*Channa diplogramma* differs from all other species in the genus by its high number of lateral line scales (103–105 vs. 36–91). It further differs from all other *Channa* species, except *C. bankanensis*, *C. lucius*, *C. micropeltes* and *C. pleurophthalma* by the presence of gular scales, a patch of scales between the anterior tips of the lower jaws, visible in ventral view. *Channa diplogramma* differs from *C. bankanensis*, *C. lucius*, and *C. pleurophtalma* by having a very different color pattern [30].

**Figure 3:**
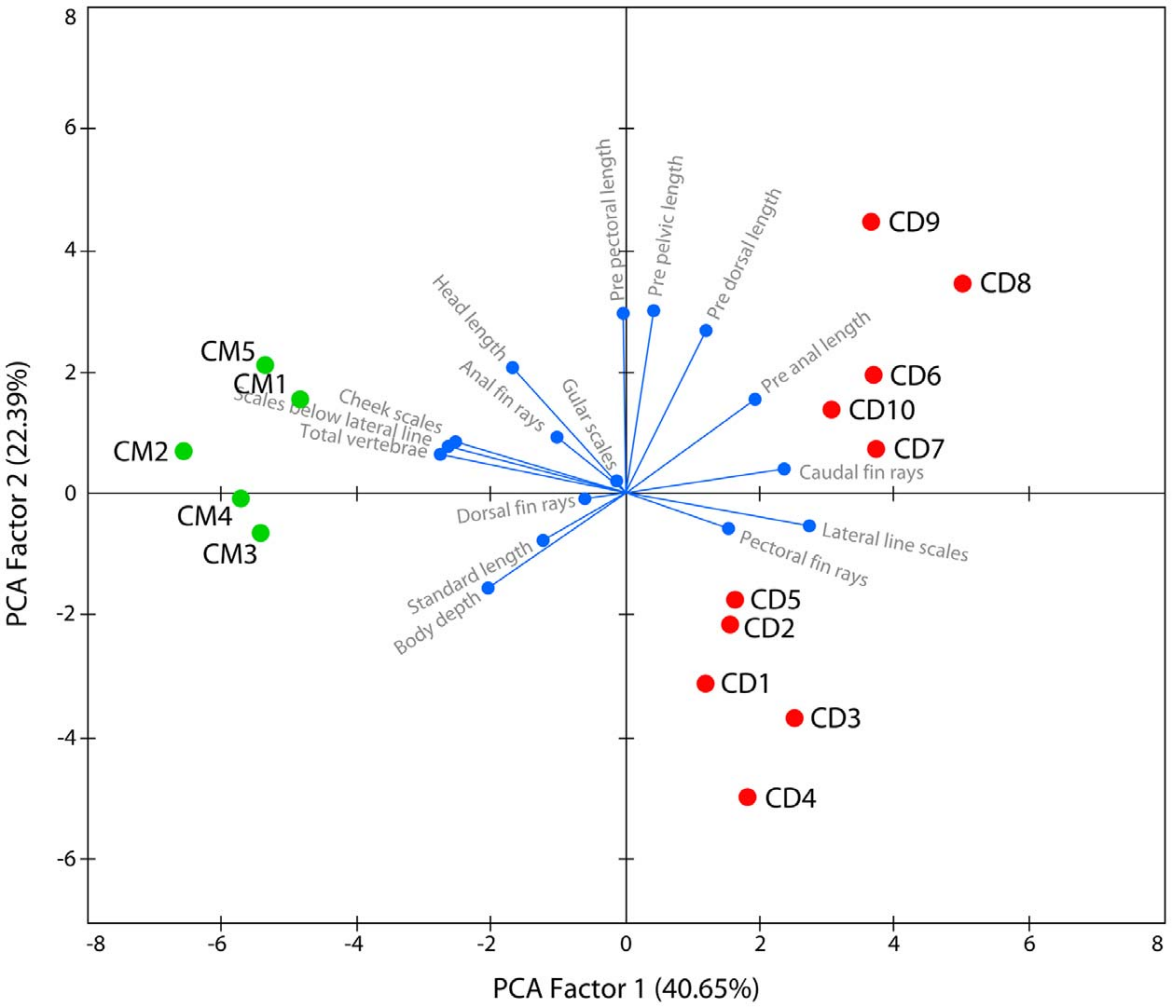
Principle Component Analysis of morphometric and meristic characters of *Channa diplogramm*a and *C. micropeltes*.

From its most closely related species, *C. micropeltes*, *C. diplogramma* can be distinguished with a combination of characters. As a percentage of standard length, pre anal length of *C. diplogramma* was significantly greater than that of *C. micropeltes* (*t* = −2.570, *df* = 13, *P* = 0.023), while body depth was significantly smaller (*t* = 2.622, *df* = 13, *P* = 0.021) (Table 1). For the meristic characters, the number of cheek scales (*t* = 8.529, *df* = 13, *P*<0.0001) and total vertebrae (one-sample *t* = −20.821, *df* = 9, *P*<0.0001) in *C. diplogramma* was significantly smaller than in *C. micropeltes*, while the number of caudal fin rays (one-sample *t* = 6.091, *df* = 9, *P*<0.0001) and lateral line scales (one-sample *t* = 72.962, *df* = 9, *P*<0.0001) was significantly higher (Table 2). PCA extracted four factors with eigenvalues higher than 1. Together, these four factors contributed to 86% of the total variation in the data. A clear separation of *C. micropeltes* and *C. diplogramma* was possible along the first PCA axis (Fig. 3). Variables, namely caudal fin rays, lateral line scales, scales below lateral line, total vertebrae, pre-anal length and body depth, had highest squared cosines on the first PCA factor.

**Table 1:** Morphometric characters of *Channa diplogramma* and *C. micropeltes*.

	*Channa diplogramma*		*Channa micropeltes*	
	Range	Mean (sd)	Range	Mean (sd)
**Total length (mm)**	107.24 (589.19)	312.45 (184.96)	338.93–654.93	502.30 (128.83)
**Standard length (mm)**	85.40 (479.15)	251.65 (151.66)	290.87–564.22	415.14 (120.11)
**% SL**				
**Head Length (mm)**	25.03 (35.37)	32.12 (2.82)	32.23–39.39	35.28 (2.64)
**Pre dorsal length (mm)**	31.47 (38.75)	35.04 (2.53)	30.50–37.57	33.25 (2.63)
**Pre pectoral length (mm)**	30.98 (38.77)	34.73 (3.26)	31.54–38.66	34.03 (3.06)
**Pre pelvic length (mm)**	31.88 (42.16)	36.93 (3.41)	34.28–41.97	37.01 (2.91)
**Pre anal length (mm)**	49.86 (60.25)	55.66 (3.42)	46.68–57.08	50.64 (3.88)*
**Body depth (mm)**	14.16 (25.61)	19.48 (3.92)	22.54–26.58	24.35 (1.68)*
**% TL**				
**Standard length (mm)**	77.14 (81.91)	79.95 (1.48)	72.42–86.15	82.29 (5.68)
**Head Length (mm)**	20.36 (27.34)	25.66 (1.99)	27.76–30.21	28.93 (0.93)**
**Pre dorsal length (mm)**	25.59 (30.86)	27.98 (1.61)	26.28–28.03	27.25 (0.64)
**Pre pectoral length (mm)**	25.13 (30.87)	27.73 (2.23)	26.66–30.52	27.89 (1.55)
**Pre pelvic length (mm)**	25.93 (32.52)	29.49 (2.28)	28.38–31.29	30.34 (1.17)
**Pre anal length (mm)**	40.55 (47.98)	44.47 (2.23)	40.06–43.64	41.51 (1.31)*
**Body depth (mm)**	11.27 (20.76)	15.60 (3.29)	17.62–22.40	20.05 (2.05)*

*P<0.05.

*P<0.01.

**Table 2:** Meristic characters of *Channa diplogramma* and *C. micropeltes*.

	*Channa diplogramma*		*Channa micropeltes*	
	Range	Mean (sd)	Range	Mean (sd)
**Dorsal fin rays**	43–44	43.20 (0.42)	43–44	43.40 (0.55)
**Pectoral fin rays**	17	17.00 (0.00)	16–17	16.60 (0.55)
**Pelvic fin rays**	6	6.00 (0.00)	6	6.00 (0.00)
**Anal fin rays**	26–28	27.50 (0.71)	27–29	28.00 (0.71)
**Caudal fin rays**	15–17	15.30 (0.67)	14	14.00 (0.00)* ^,^ a
**Lateral line scales**	103–105	104.20 (0.79)	86	86.00 (0.00)* ^ ,^ a
**Cheek scales**	16–20	17.80 (1.55)	23–25	24.20 (0.84) *
**Gular scales**	30–31	30.60 (0.52)	18–39	30.60 (10.26)
**Total vertebrae**	53–54	53.60 (0.52)	57	57.00 (0.00)* ^ ,^ a

*P<0.0001.

a one sample t test.

### Redescription

#### Large species, reaching a maximum length of at least 480 mm standard length (SL)

Body elongated. Body depth is 14.2–25.6% of SL. Cross section of body is circular in anterior portion, somewhat compressed posteriorly in the caudal area. Body depth is greatest at insertion of dorsal fin. Body width is greatest at insertion of pectoral fin (11.18–21.62% of SL). Head is large, long (25.02–35.06% of SL), dorsally flattened and rounded anteriorly, covered by scales anteriorly up to level of posterior nostrils. Head depth is 52.0–69.3% of head length (HL). Head width is 63.45–86.75% of HL. Inter-orbital region narrow (25.20–40.86% of HL) and slightly convex. Eye diameter 10.12–20.83% of HL. Mouth large, upper jaw length 37.9–51.6% of HL, maxilla extending posteriorly beyond posterior margin of eye. Predorsal scales 21–23. Gular portion covered with 30–31 gular scales. Cephalic sensory pores open via numerous satellite openings in the skin.

#### Scales on head and body small

Cheek scales 16–20. Lateral line scales small, 103–105. Scale rows above lateral line 10.5, below lateral line 15. Circumpeduncular scales 15–16. Dorsal fin rays 43–44. Anal fin rays 26–28. Pectoral fin rays 17. Pelvic fin with 6 rays. Principal caudal fin rays 15–17. Total vertebrae 53–54. Outer margins of pectoral and caudal fins rounded.

#### Mouth is big, terminal, with maxilla reaching anteriorly slightly posterior to a vertical through anterior nostril

Many rows of small conical teeth on premaxilla, an additional series of 2–3 times larger conical teeth anteromedially on the premaxilla. Several rows of small teeth at the symphysis, numbers of rows and size of teeth decreasing ventrally along the pre-maxilla towards its posteroventral tip. Vomer and palatine with a series of small teeth marginally, followed medially by several conspicuous, large canines. Dentary with a marginal row of large teeth restricted to the area close to the symphysis, followed medially by several rows of small teeth extending along the dentary and an internal row of conspicuous, large canines. Many variously sized conical teeth on vomer and palatine, those on inner row much larger and canine-like.

### Coloration

In life (see subsequent section on ontogenic color phases).

### Distribution

*Channa diplogramma* is endemic to the southern Western Ghats of peninsular India. It is known from the Rivers (including its principal reservoirs) Meenachil, Manimala, Pampa, Achenkovil and Kallada in Kerala state, as well as the Chittar and Tambraparini Rivers (and its reservoirs) in Tamil Nadu state (see Fig. 1).

### Ontogenic color phases of Channa diplogramma

**Figure 4:**
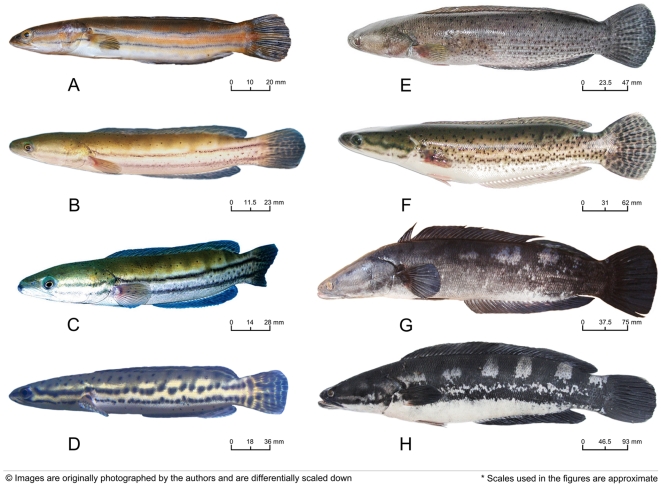
Ontogenetic color phases of *Channa diplogramma.* A: Fingerling; B: Fingerling, C: Juvenile, D: Juvenile, E: Sub-Adult, F: Sub-Adult, G: Adult, H: Adult (length in millimeters is given as a scale below each specimen); all individuals were collected from the river Meenachil in Kerala, India.

*Channa diplogramma* shows multiple color phases during its life history (Fig. 4), which makes local fishers, believe that they are different species. The different specimens are also known by different vernacular names (*Pulivaka, Karivaka, Manalvaka, and Charalvaka*). We collected eight differently colored specimen (Fig. 4) of *C. diplogramma* from the rivers Pamba and Meenachil in Kerala, India, which occur sympatrically and utilize the same ecological habitat. *Channa micropeltes* also possess similar ontogenic color phases [51] like *C. diplogramma*. However, local knowledge of the fishers in the Mekong River attributes this color variation of *C. micropeltes* to the differential habitat occupancy of the individuals [51]. Due to logistical difficulties, we were unable to obtain all the morphs of *C. micropeltes* for the present study.

We did not observe any individual of the Malabar Snakehead measuring less than 97.1 mm TL and so do not have any information on the color pattern or external morphology of early larvae and fry of *C. diplogramma*. In fingerlings and early juveniles, a broad black band passes through the eye straight to the upper half of the caudal fin (Fig. 4; A–D), and a second black line commences at the angle of the mouth, and proceeds to the lower half of caudal region. An orange colored stripe passes in between these black bands, and the orange color covers most of the dorsal region. During subsequent development (large juveniles), the orange stripe fades and becomes yellow to light brown, and light black; later the black lines fade and black colored spots appear on the body (Fig. 4; E–F), which changes the color then to off white and grey. From the sub-adult stage, the black colored spots coalesce and four to six white blotches appear on the sides of the body starting from the dorsum downwards up to the lateral line region, later becoming conspicuous in adults (Fig. 4; G–H). In large adults, the abdomen is pure white, the caudal fin, dorsal surface, cheeks and head in general are black, with a purple tint, while dorsal and anal fins have a grey border.

The ten individuals of *C. diplogramma* used for morphometric and meristic character assessment (Tables 1 and 2) included all the range of color morphs previously described (two individuals each of morphs A and H, and one sample each of morphs B, C, D, E, F and G; see Fig. 4). All these ten individuals have almost identical morphometric and meristic characters. Our analyses of the COI and 16S rRNA gene sequences from different color phases of *C. diplogramma* (morphs A, C, D, E, G and H; see Fig. 4) also revealed that they are genetically identical (same molecular profile; see Table S2 for details).

### Phylogenetic relationships

**Figure 5:**
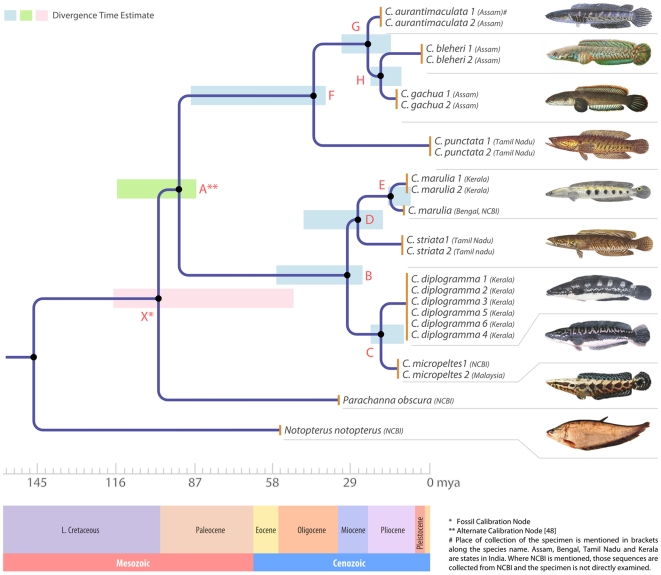
Phylogram showing the relationships of the channids used in this study rooted with *Notopterus notopterus* (AP008925.1). The nodes for which the divergence time is presented in tables 3 and 4 are labeled as A through H below the branches; the mean time intervals of divergence calculated by the two calibration methods are represented as rectangular bars on the nodes.

The 36 nucleotide sequences of the Indian channids (six sequences each of 16S rRNA and COI for *C. diplogramma* and two 16S rRNA and two COI sequences each for the other six channids used in the study) were submitted to GenBank (Accession Numbers: EU342175 to EU342210; Table S2). In addition, one sequence each of COI and 16S rRNA from the specimen of *C. micropeltes* used in the present study has been submitted to NCBI (Accession No: JF900369 and JF900370). The phylogenetic trees constructed using the Maximum Likelihood method yielded well-resolved phylogenies in all the cases. GTR+G+I was found to be the best-fit nucleotide substitution model for both the mtDNA 16S rRNA and COI genes. A phylogenetic tree constructed with the 16S rRNA gene sequences, including a sequence of *C. micropeltes* by Smith and Wheeler; DQ532852) [52], *C. marulius* from North East India [53] and *Parachanna obscura* (AY763726), along with the sequences that we generated, clearly distinguishes *C. diplogramma* from *C. micropeltes* (90% bootstrap support; Fig. S1). Similarly, the two species were clearly differentiated in the phylogenetic tree based on the COI sequences (99% bootstrap support; Fig. S2). The concatenated dataset produced a similar topology (Fig. 5) with high bootstrap support values for all clades. The results of our genetic distance calculations showed that *C. diplogramma* and *C. micropeltes* showed the highest intra-specific genetic distance (2.4–3.0% for 16S rRNA and 21% for COI; Table S3 and S4), yielding support that *C. diplogramma* is a separate species concordant with the morphometric analysis.

### Divergence time estimates

The divergence time for *C. diplogramma* and *C. micropeltes* was calculated as 7.77 MYA using fossil calibration, and 17.68 MYA with the alternate calibration in the LMRD method (assumes local rates for every internal node and it is used when the sequence dataset is assumed to be not clock-like). The mean divergence time values for the node E that correspond to the split between *C. marulius* from North East India and South India (see Fig. 5) was 6.56 and 15.00 MYA with the two different calibrations, which are very high divergence values for individuals from the same species. The high genetic divergence and divergence time estimates between *C. marulius* from geographically isolated locations points towards the presence of further cryptic species within the genus *Channa* that should be investigated using comprehensive sampling and detailed taxonomic and genetic analyses. The results of the tree calibrations (Fig. 5) are presented in Tables 3 and 4.

**Table 3:** Results of divergence time estimation in million years for the various nodes of the phylogenetic tree presented in Figure 5 the calibration point at node X was the earliest channid fossil age from Eocene (∼50 MYA; [51]).

Node	LMRD	GMRD	NPRS	NPRS-Log	Mean divergence time
**X** *	50	50	50	50	50
**A**	40.49	41.72	43.35	40.25	41.425
**B**	24.09	24.04	28.27	21.56	24.49
**C**	7.77	8.5	13.9	7.914	9.52
**D**	19.19	19.74	24.14	17.67	20.185
**E**	5.317	6.349	9.26	5.301	6.556
**F**	38.1	37.63	40.27	36.32	38.08
**G**	10.65	13.64	22.42	11.13	14.46
**H**	5.633	8.866	16.67	6.781	9.4875

*calibration node.

**Table 4:** Results of divergence time estimation in million years for the various nodes of the phylogenetic tree presented in Figure 5 the calibration point at node one was the split between Parachanna and Channa calculated by Li et al., (110-84 MYA) [48].

Node	LMRD	GMRD	NPRS	NPRS-Log	Mean divergence time
**X**	120	116.2	115.2	120.6	118
**A** *	110-84	110-84	110-84	110-84	110-84
**B**	56.29	55.86	62.19	52.20	56.64
**C**	17.68	19.76	30.34	19.25	21.76
**D**	44.44	45.88	52.96	42.78	46.52
**E**	12.25	14.76	20.17	12.84	15.00
**F**	91.01	87.44	89.79	87.63	88.97
**G**	24.68	31.72	49.66	26.82	32.22
**H**	12.85	20.61	36.87	16.33	21.67

*Calibration node.

## Discussion

After Francis Day's (1865) [15] initial description of *C. diplogramma* he himself synonymised the species with *C. micropeltes* in 1878 [17]. Since then, there have been no collections of *C. diplogramma* for detailed taxonomic investigations, and all subsequent information in the literature [9], [18]–[20], [22]–[26] was based on Day's (1878) synonymy [17]. The highly fragmented distribution of *C. micropeltes* and its markedly different adult appearance (with the individuals in peninsular India), based on observation in various public and retail aquariums (Ralf Britz; Rajeev Raghavan Pers. Observation), led us to examine the systematic position of the species in detail.

Color pattern is frequently used as the sole character to distinguish closely related species. This is well justified if it serves as a primary cue in the recognition of con-specifics [54]–[55]. However, using coloration as a basis for species identification may turn problematic if color variation is a result of phenotypic plasticity, rather than reproductive isolation [56]. Another concern is that coloration genes [57] may evolve more rapidly [58] than other morphological and genetic characters. Channids are well known for the fact that the color patterns of their juveniles are very different from that of the adults [59], although the reasons for this difference remain unknown. During the life history of *C. diplogramma*, individuals have multiple color phases. However, it was observed that these individuals, belonging to different life stages, of *C. diplogramma* occur sympatrically and utilize the same ecological habitat, unlike the observations from South East Asia, where local knowledge of fishers reveals that the color variation in *C. micropeltes* is linked to the differential habitat occupancy by the individuals [51].

The gular scales [30], a morphological trait that has been hypothesized to be plesiomorphic [51] at the level of the family Channidae has been reported only in four species of channids endemic to South East Asia, *C. bankanensis*, *C. lucius, C. micropeltes*, and *C. pleurophthalma*, apart from the *Parachanna* of Africa [30], [51]. Our observation of gular scales in *C. diplogramma* makes it the only species of channid from the Indian subcontinent with gular scales, a character shared with its sister species *C. micropeltes* (Fig. S3).

The morphometric and meristic analysis of *C. micropeltes* and *C. diplogramma* provided conclusive evidence to separate them as two distinct species. Our analyses indicate that number of caudal fin rays, lateral line scales, scales below lateral line; total vertebrae, pre-anal length and body depth were the most prominent characters that can be used to differentiate both the species.

A high genetic differentiation at the intraspecific level was observed for *C. marulius* (2.1% for the 16S rRNA gene) that included individuals from Bengal, North East India [53] and from Kerala, South India (present study). All the other species showed lower intraspecific genetic differentiation values. The genetic distance between *C. micropeltes* (sequence [52] and *C. micropeltes* present study) and *C. diplogramma* from South India (present study) was 2.7–3.0% (for 16S rRNA gene sequence comparison), and the genetic distance for the COI gene sequences were 21% between these species - which was well above the average observed for any other intraspecific genetic distances (table S3 and S4). This indicates that *C. micropeltes* and *C. diplogramma* cannot be considered conspecific, and results of both morphological and genetic analyses clearly support the existence of two distinct species.

Recent studies have estimated the molecular divergence time dates for channids. Some researchers [48] have favored the hypothesis that a vicariant divergence of channids occurred during the Gondwanaland split based on a divergence time calibration using reliable biogeographic scenarios and fossil records. By contrast, others [14] favored the “out of Asia into Africa” hypothesis when calibrating the tree solely based on fossil records. In this study, we calibrated the phylogenetic tree with two alternative constraints, one based on the oldest known fossil of channids and the other based on the available molecular divergence time estimate for the emergence of the genus *Channa*. Due to the incomplete nature of the fossil record, fossil calibrations can only provide minimum ages and therefore, will tend to underestimate lineage divergence times [60]. To reduce such bias we calibrated the tree a second time with a previously calculated value of 110–84 MYA for the mean divergence time of the emergence of the genus *Channa* [48]. This divergence time value was attained based on the continental breakup of African and South American landmasses (100–120 MYA) and the estimated divergence time between sarcopterygians and actinopterygians (420–500 MYA), which has been successfully used previously to date old divergence times in actinopterygian fishes [61]–[62]. Moreover, the recent identification of channid fossils from Africa in the middle Eocene [63], further supports the use of this additional time constraint, and highlights the incomplete nature of the fossil records.

The fossil records (including the oldest known channid fossil) from Northwest Pakistan had faunal affinities towards both Asia and Africa [64], which could be due to the contact, of the drifting Indian subcontinent, with Africa, during its northward movement allowing the dispersion of African fauna into Asia [65]. Thus, assigning a center for the origin of channids in the Indian subcontinent could be erroneous. We therefore speculate a vicariant divergence of *Parachanna* and *Channa* genera during the Gondwana land breakup, with the genus *Channa* dispersing into Eurasia. It is likely that fishes of the genus *Channa* could have been widely distributed from South East Asia to the Indian Subcontinent (or vice versa) during the multiple contacts of the two land masses [66]–[68] during the drift to the present positions.

Our average divergence time estimates between *C. diplogramma* and *C. micropleltes* were from ∼9.52 (with fossil data) to ∼21.76 MYA (with the alternative calibration). According to the Satpura Hypothesis [69], the westward migration of Malayan fishes deflected southwards in the late Miocene (∼10–15 MYA) due to the formation of a ridge in the North (the Nepal Ridge) of the Himalayas. Thus, our lower values attained by fossil calibration for the split of *C. diplogramma* and *C. micropeltes* are in concordance with this time frame of migration of fishes from Malaya. However, this may only hold good for torrential freshwater fishes, and the dispersion of channids through this route could be difficult to explain. The mean upper value of ∼21.76 MYA (early Miocene) makes it highly improbable for this species to have dispersed towards India from South East Asia, or having originated in Northwest India, due to the absence of any geographic connections towards Southern India during this time frame. Another scenario is the dispersal of the most recent common ancestor of these two species from Southern India through North East India to South East Asia, in a reverse direction. However, this scenario can be ruled out due to the above said reasons. Thus, the Satpura hypothesis or the origin of the most recent common ancestor of *C. diplogramma* and *C. micropletes* in Northwestern India cannot conclusively explain the presence of *C. diplogramma* in peninsular India.

Hence, the most plausible scenario for the evolution of channids would be a vicariant divergence after the Gondwanaland split-up, of the genus *Parachanna* into Africa and the genus *Channa* into Eurasia. The presence of *C. diplogramma* in South India, also point towards a scenario of the vicariant divergence of the most recent common ancestor of *C. micropeltes* and *C. diplogramma* during the drift of the South East Asian and Indian sub-continental land masses towards its present positions [66]–[68].

Our study clearly supports the recognition of *C. diplogramma* as an endemic species of peninsular India, subsequently justifying its high conservation value due to its restricted distribution. Like all channids, *C. diplogramma* is a ‘K selected’ species with a slow growth, long time to reproduce and longer life, which makes them highly vulnerable to overexploitation [27]. *Channa diplogramma* is a connoisseurs' delight in Central Kerala and locals pay premium prices for sub adult and adult specimens. Local fishers operating in the rivers and reservoirs where this species is known to occur have confirmed its rarity and that populations have declined considerably (> 90%) over the last two decades.

In addition to the indiscriminate exploitation by local fishers, *C. diplogramma* is also severely threatened by the loss of critical riverine habitats due to sand mining and reclamation of riverine areas for the construction boom in Kerala, as well the increasing pollution in existing habitats due to domestic and industrial sewage.

The key to effectively preserving the remaining populations of *C. diplogramma* will therefore need to consider: (i) habitat protection, (ii) fishery management plans (regulation of total allowable catch, restrictions on mesh sizes and closed seasons), and (iii) the development of a captive breeding technology for facilitating large scale ranching and stock enhancement in the rivers and reservoirs where the species occur.

The International Union for Conservation of Nature (IUCN) has recently completed a comprehensive assessment of freshwater biodiversity in the Western Ghats Hotspot. However, the Western Ghats species list does not include *C. diplogramma* as it is still considered to be a synonym of *C. micropeltes* in the Catalog of Fishes [50], the database from which the species list were compiled. The experts at the IUCN Workshop including two of the authors of this paper have however suggested that the “Indian race” of *C. micropeltes* should be considered as distinct and its conservation status categorized as ‘Vulnerable’.

### Conclusion

The species status of *C. diplogramma* as an endemic species of peninsular India has been confirmed through both morphological and molecular analyses after a period of 146 years since its initial description, and 134 years after it was synonymised. Our results suggest that this species shared a most recent common ancestor with *C. micropeltes*, around 9.52 to 21.76 MYA. An effective conservation effort specifically targeted for this enigmatic and economically important species is highly recommended to avoid endangerment and possible extinction in its restricted range. Also, there is a need for carrying out comprehensive taxonomic and genetic profiling of the Snakeheads in tropical Asia to identify its population structure, and also to evaluate the likelihood of additional species. This is of utmost importance as the Snakeheads are widely exploited as food and ornamental fishes, and their conservation and management is a priority in many Asian countries where their populations are declining.

## Supporting Information

Figure S1 **Phylogenetic tree of the channid species used in the study with partial mitochondrial 16S rRNA gene sequences, rooted with** ***Notopterus notopterus*** **.** Bootstrap values below 60 are not shown.
(PNG)

Figure S2 **Phylogenetic tree of the channid species used in the study with partial mitochondrial COI gene sequences, rooted with** ***Notopterus notopterus*** **.** Bootstrap values below 60 are not shown.
(PNG)

Figure S3 **Photographs showing the gular scales of** ***C.diplogramma*** **(left) and** ***C. micropeltes*** **(right).**
(PDF)

Table S1 **Morphometric and meristic measurements of the specimen used in this study.** The table contains measurements of the holotype of *C. diplogramma* (BMNH 1865.7.17.24 unique Holotype; NMW 73835, NMW 73838 and NMW 84220 Day's specimen) and Syntpes of *C. micropeltes* (RMNH D2318 Syntype; RMNH D1131 possible Syntype).
(PDF)

Table S2 **List of fish species used for the study, their NCBI accession numbers, voucher numbers and the respective museums.**
(PDF)

Table S3 **Genetic distance values calculated for the partial mitochondrial COI sequences of different Channa species used in the study.**
(PDF)

Table S4 **Genetic distance values calculated for the partial 16s rRNA gene sequences of different Channa species used in the study.**
(PDF)
