# Supplementary figures and images for: Unraveling a 146 Years Old Taxonomic Puzzle: Validation of Malabar Snakehead, Species-Status and Its Relevance for Channid Systematics and Evolution
Source: PLoS One. 2011 Jun 24;6(6):e21272. doi: 10.1371/journal.pone.0021272 (PMC3123301; doi:10.1371/journal.pone.0021272)

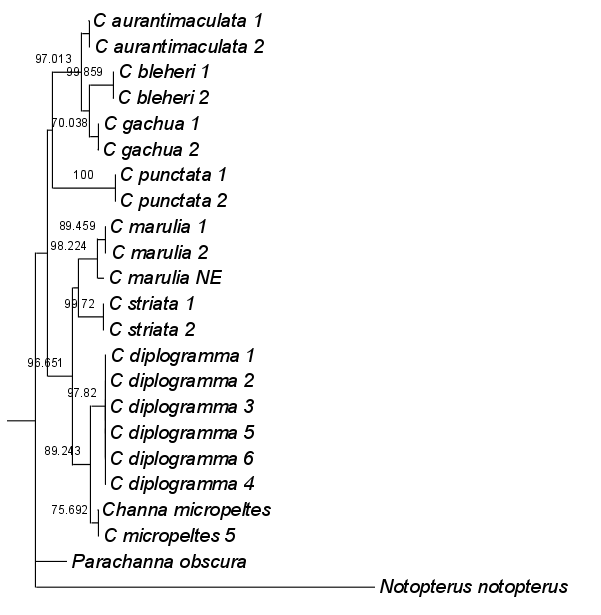

Supplement: Figure S1 — Phylogenetic tree of the channid species used in the study with partial mitochondrial 16S rRNA gene sequences, rooted with Notopterus notopterus . Bootstrap values below 60 are not shown. (PNG) [file pone.0021272.s001.png]

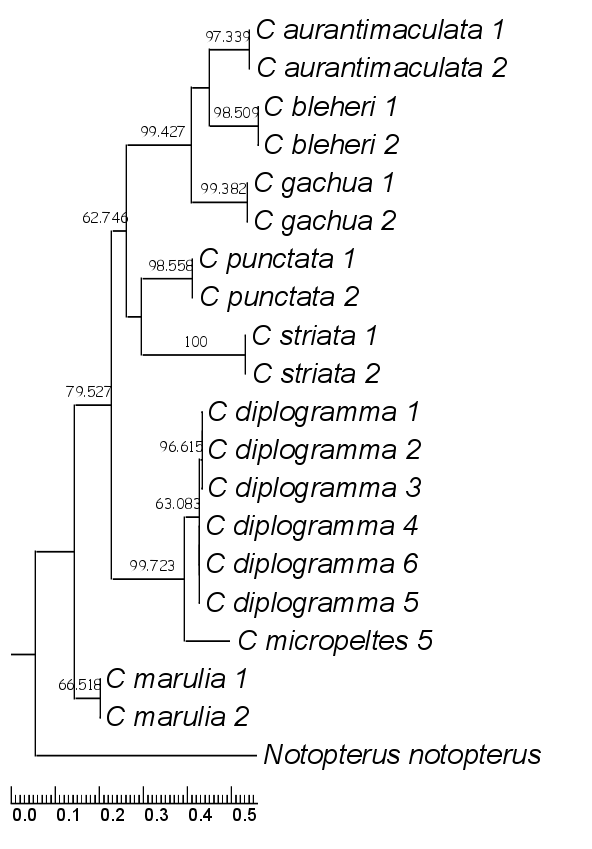

Supplement: Figure S2 — Phylogenetic tree of the channid species used in the study with partial mitochondrial COI gene sequences, rooted with Notopterus notopterus . Bootstrap values below 60 are not shown. (PNG) [file pone.0021272.s002.png]

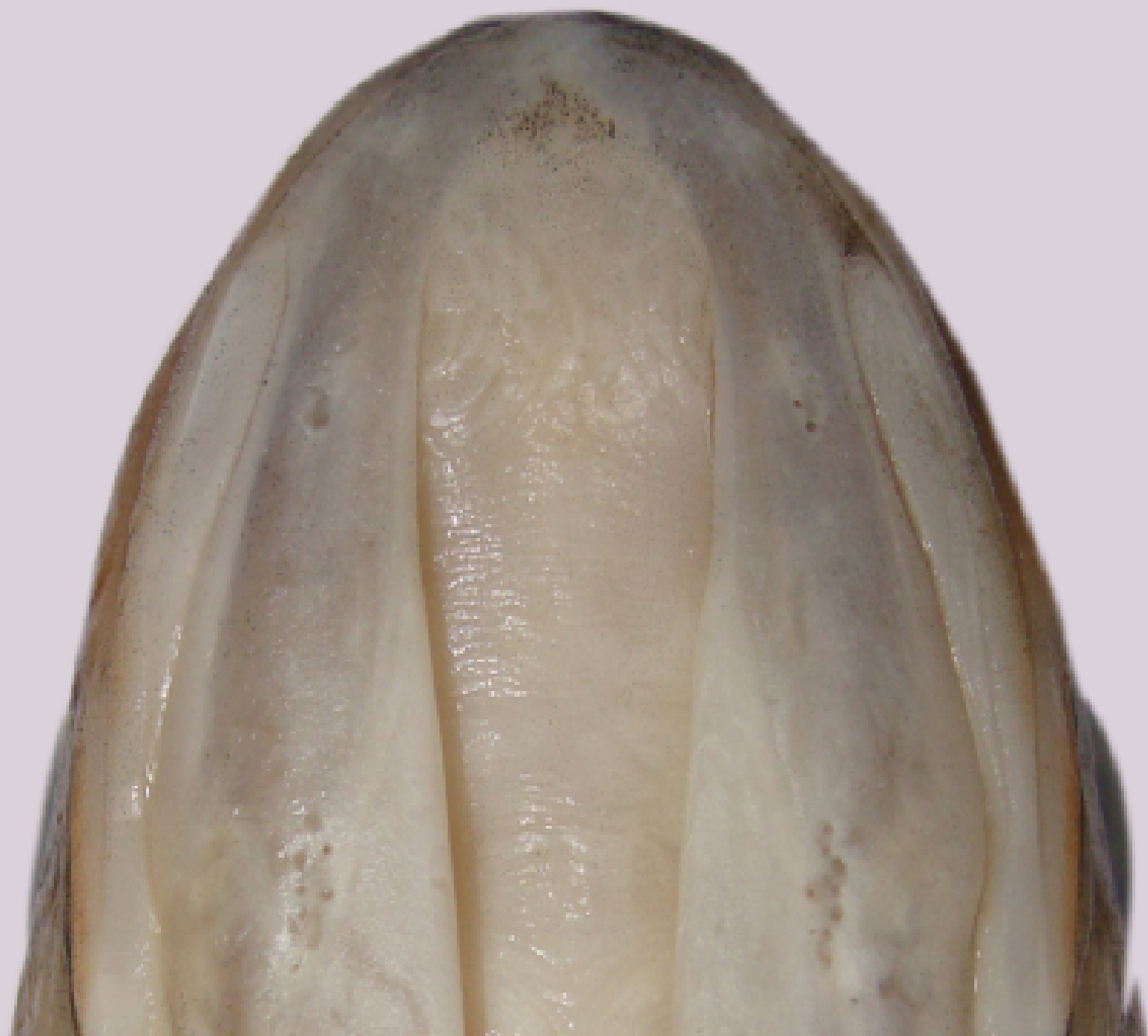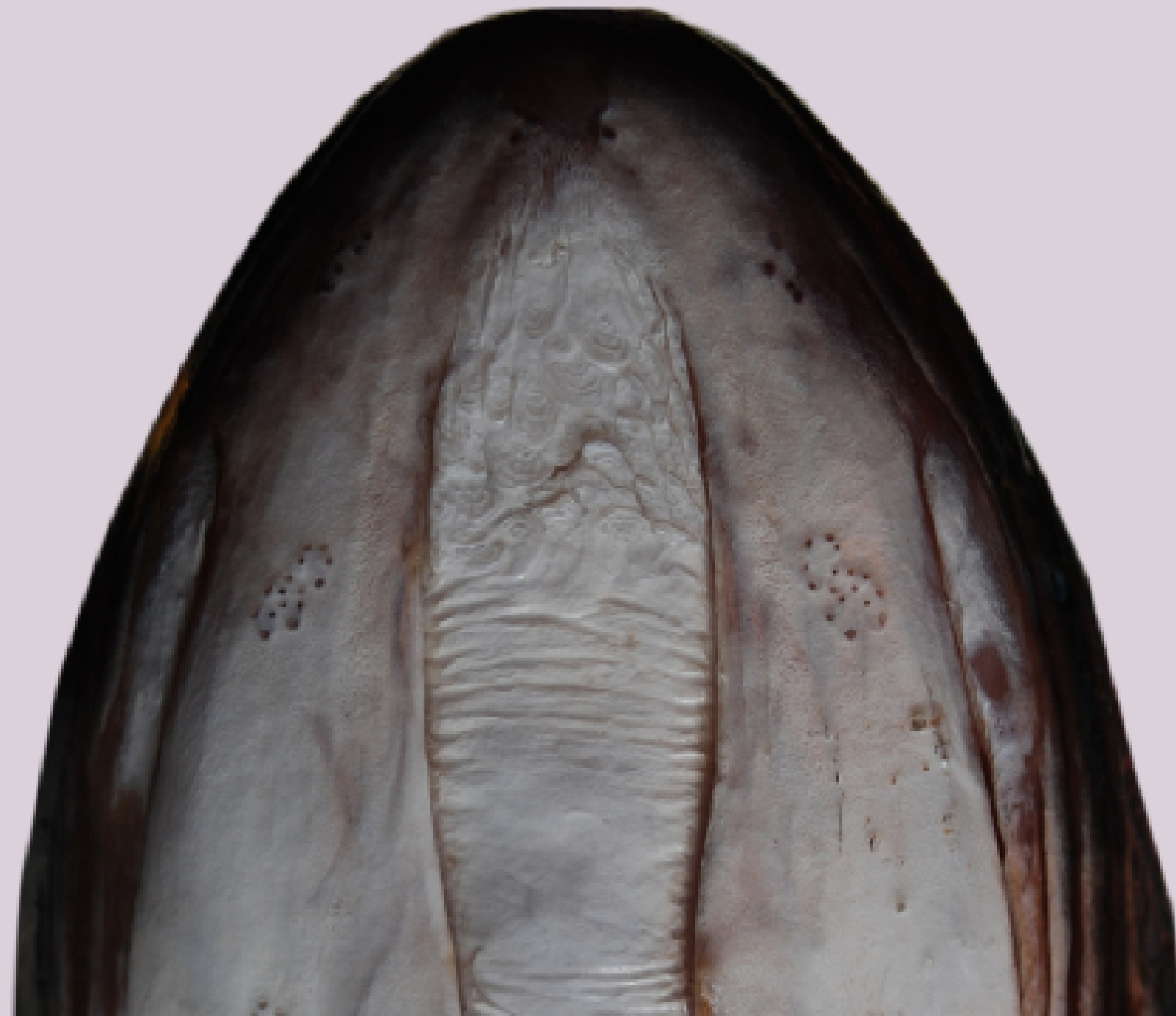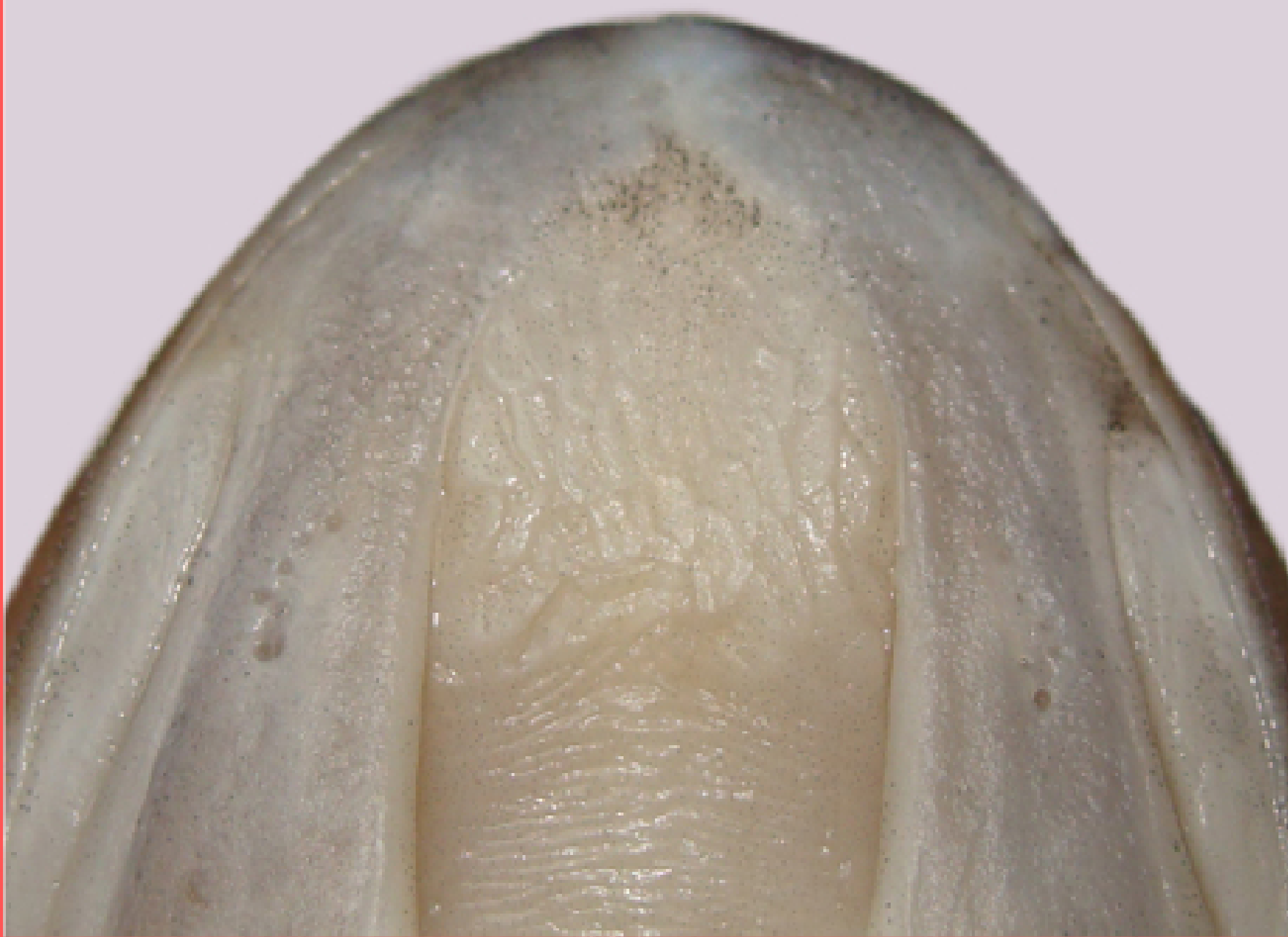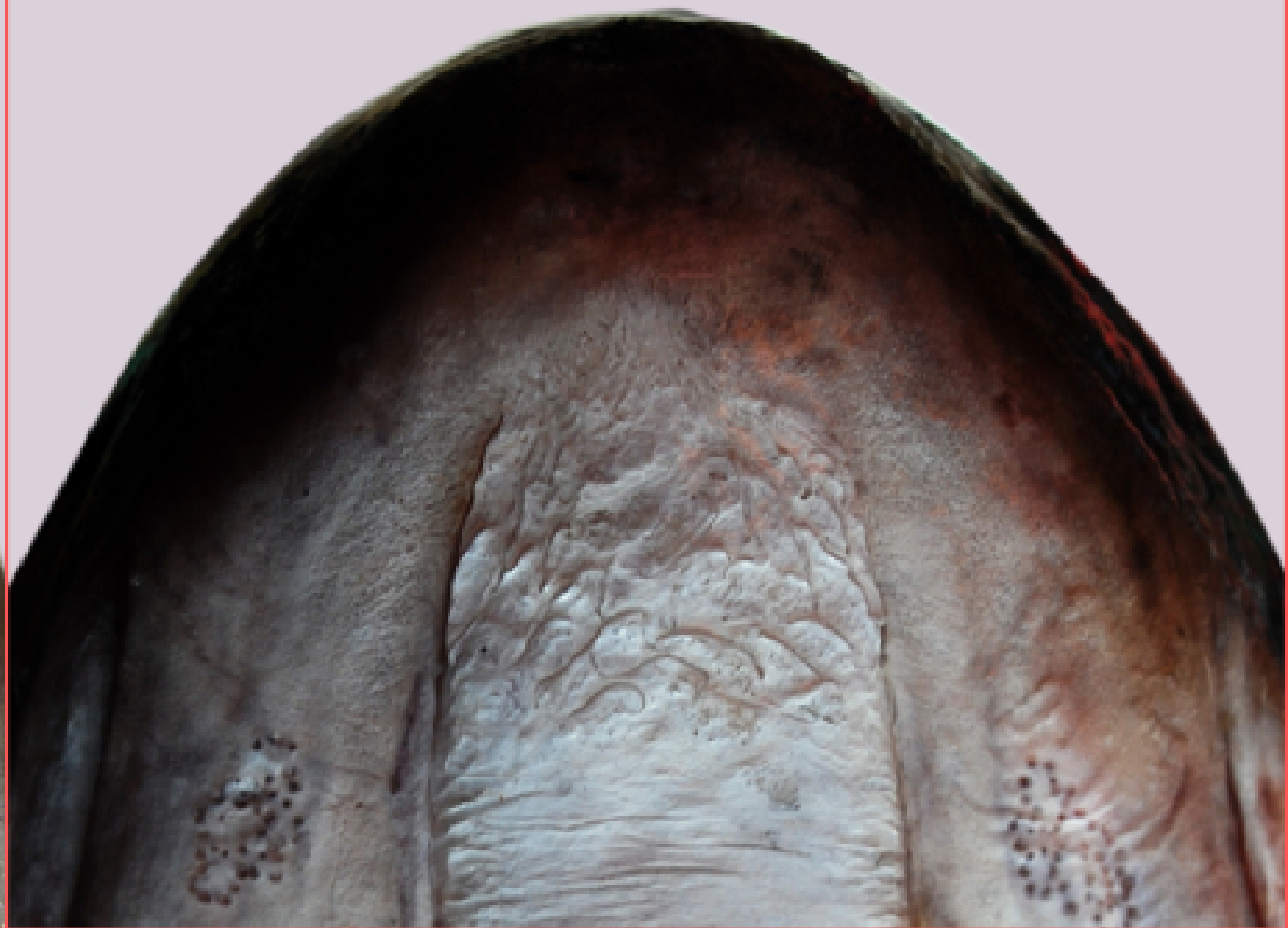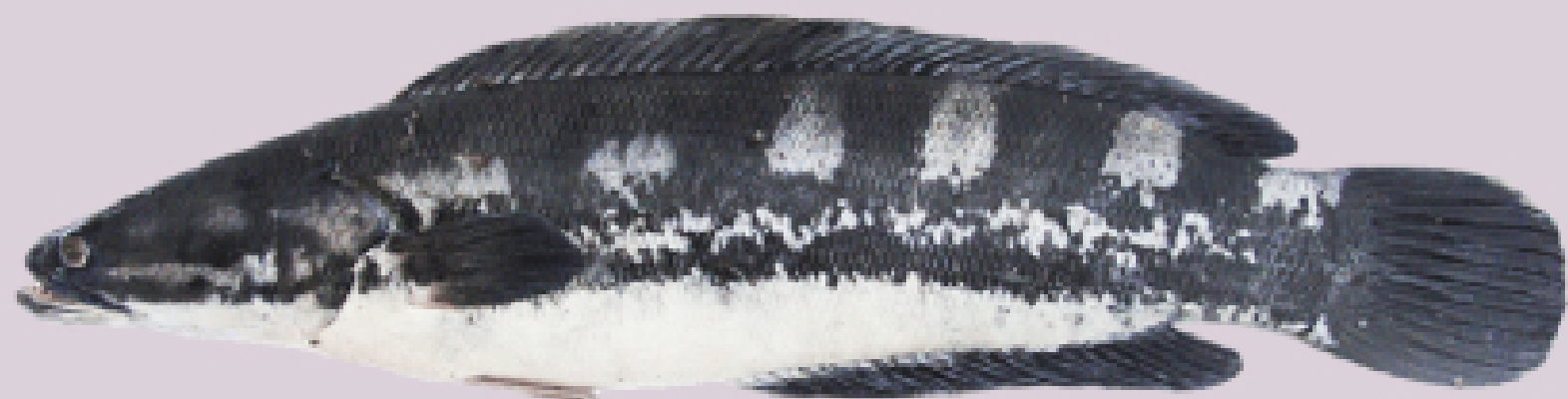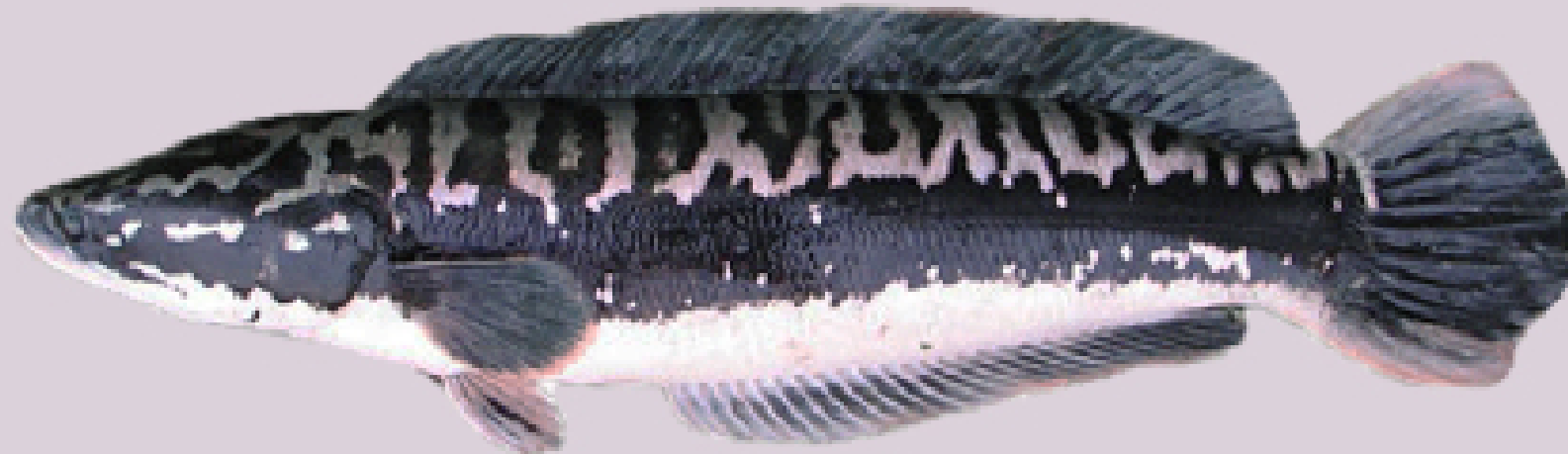

Supplement: Figure S3 — Photographs showing the gular scales of C.diplogramma (left) and C. micropeltes (right). (PDF) [file pone.0021272.s003.pdf]
