# Supplementary material for: Unraveling a 146 Years Old Taxonomic Puzzle: Validation of Malabar Snakehead, Species-Status and Its Relevance for Channid Systematics and Evolution
Source: PLoS One. 2011 Jun 24;6(6):e21272. doi: 10.1371/journal.pone.0021272 (PMC3123301; doi:10.1371/journal.pone.0021272)
Supplement: Table S1 — Morphometric and meristic measurements of the specimen used in this study. The table contains measurements of the holotype of C. diplogramma (BMNH 1865.7.17.24 unique Holotype; NMW 73835, NMW 73838 and NMW 84220 Day's specimen) and Syntpes of C. micropeltes (RMNH D2318 Syntype; RMNH D1131 possible Syntype). (PDF) [file pone.0021272.s004.pdf]

Table S1: Morphometric and meristic measurements of the specimen used in this study; the table contain measurements of the holotype of *C. diplogramma* (BMNH 1865.7.17.24 unique Holotype; NMW 73835, NMW 73838 and NMW 84220 Day's specimen) and Syntpes of *C. micropeltes* (RMNH D2318 Syntype; RMNH D1131&D1132 possible Syntypes); measurements are in millimeters.

	*UMT CM#1	UMT CM2	UMT CM3	UMT CM4	UMT CM5					
	***Channa micropeltes* fresh specimen measured in this study**									
Total length (mm)	433.35	477.03	607.24	654.93	338.93					
Standard length (mm)	313.82	394.91	511.88	564.22	290.87					
Body weight (g)	800	1200	2890	3300	355					
Head Length (mm)	123.61	140.48	174.22	181.82	102.39					
Pre dorsal length (mm)	117.91	131.31	165.1	172.1	95.01					
Pre pectoral length (mm)	121.32	129.3	161.89	177.95	103.45					
Pre pelvic length (mm)	131.72	135.37	185.06	204.12	106.05					
Pre anal length (mm)	179.12	195.82	251.83	285.79	135.78					
Body depth (mm)	76.37	89.01	136.05	143.18	66.74					
Dorsal fin rays	43	43	44	44	43					
Pectoral fin rays	16	16	17	17	17					
Pelvic fin rays	6	6	6	6	6					
Anal fin rays	28	29	27	28	28					
Caudal fin rays	14	14	14	14	14					
Lateral line scales	86	86	86	86	86					
Scales below lateral line	16	16	16	16	16					
Cheek scales	24	25	24	25	23					
Gular scales	21	39	18	36	39					
Total vertebrae	57	57	57	57	57					
	***CRG-CHDIP# 20**	**CRG-CHDIP 21**	**CRG-CHDIP 22**	**CRG-CHDIP 23**	**CRG-CHDIP 24**	**CRG-CHDIP 25**	**CRG-CHDIP 26**	**CRG-CHDIP 27**	**CRG-CHDIP 28**	**CRG-CHDIP 29**
	***Channa diplogramma* fresh specimen measured in this study**									
Total length (mm)	445.56	328.21	488.88	589.19	525	148.56	189.72	129.75	107.24	172.39
Standard length (mm)	361.16	266.6	389.69	479.15	430.05	116.53	149.92	100.09	85.4	137.88
Body weight (g)	1200	400	1200	1700	160	234	541	149	78	405
Head Length (mm)	118	83.71	125.06	119.93	132.41	38.74	50	35.4	29.32	45.45
Pre dorsal length (mm)	118.72	91.11	132.66	150.8	138.92	43.59	53.64	38.42	33.09	48.53
Pre pectoral length (mm)	111.95	85.3	123.59	148.42	141.88	44.24	55.08	38.28	33.11	50.84

Pre pelvic length (mm)	125.65	89.64	133.14	152.76	151.75	46.8	57.23	42.2	34.17	53.82
Pre anal length (mm)	187.49	142.8	210.13	238.89	240.68	68.39	83.8	60.08	51.45	77.98
Body depth (mm)	92.48	60.29	97.79	84.63	91.44	20.57	26.25	15.86	12.09	24.04
Dorsal fin rays	43	43	43	43	44	44	43	43	43	43
Pectoral fin rays	17	17	17	17	17	17	17	17	17	17
Pelvic fin rays	6	6	6	6	6	6	6	6	6	6
Anal fin rays	28	28	26	28	27	27	27	28	28	28
Caudal fin rays	15	15	15	15	15	15	16	17	15	15
Lateral line scales	103	104	104	103	105	105	104	105	104	105
Scales below lateral line	15	15	15	15	15	15	15	15	15	15
Cheek scales	16	18	16	18	20	18	19	17	20	16
Gular scales	30	31	31	31	30	31	31	30	30	31
Total vertebrae	53	54	53	54	54	53	53	54	54	54
	***BMNH 1865.7.17.24**	***NMW 73835**	**NMW 73838**	**NMW 84220**						
	***Channa diplogramma* type specimen measured in this study**									
Total length (mm)	97.1	424	275	459						
Standard length (mm)	81.6	352	230	380						
Body weight (g)	---	114	76.8	125						
Head Length (mm)	28.4	109	77.5	120.5						
Pre dorsal length (mm)	29.5	113.5	86.1	117.2						
Pre pectoral length (mm)	29.1	120	114	131						
Pre pelvic length (mm)	29.6	171.6	46.7	185						
Pre anal length (mm)	43.8	65.7	---	68.8						
Body depth (mm)	11.9	---	---	---						
Dorsal fin rays	44	44	45	43						
Pectoral fin rays	18	18	18	18						
Pelvic fin rays	6	6	6	6						
Anal fin rays	28	28	28	27						
Caudal fin rays	14	14	14	14						
Lateral line scales	---	106	107	106						
Scales below lateral line	---	20	21	20						
Cheek scales	21	20	22	19						
Gular scales	---	---	---	---						

Total vertebrae	55	---	---	---
	***RMNH D2318**	**RMNH D1131**	**RMNH D1132**	
	***Channa micropeltes* type specimen measured in this study**			
Total length (mm)	710	261	301	
Standard length (mm)	605	210	250	
Body weight (g)	---	---	---	
Head Length (mm)	185	59	---	
Pre dorsal length (mm)	---	---	---	
Pre pectoral length (mm)	---	---	---	
Pre pelvic length (mm)	---	---	---	
Pre anal length (mm)	---	---	---	
Body depth (mm)	---	---	---	
Dorsal fin rays	44	43	43	
Pectoral fin rays	17	17	17	
Pelvic fin rays	6	6	6	
Anal fin rays	27	28	28	
Caudal fin rays	14	14	14	
Lateral line scales	---	---	---	
Scales below lateral line	---	---	---	
Cheek scales	---	---	---	
Gular scales	---	---	---	
Total vertebrae	---	---	---	

***BMNH** – Natural History Museum, London, United Kingdom; ***RMNH** - Rijksmuseum van Natuurlijke Histoire RMNH/Naturalis, Leiden, The Netherlands; ***NHM** – Natural History Museum, Vienna, Austria; ***UMT** – Universiti Malaysia Terengganu, Kuala Terengganu, Malaysia; ***CRG**- Conservation Research Group, Department of Aquaculture, St. Albert's College, Kochi, India.

#Voucher specimens of *C. diplogramma* examined in this study are currently deposited at the museum of CRG, Department of Aquaculture. St. Albert's College, Kochi, India (CRG-CHDIP-20-CRG-CHDIP- 29), while those of *C. micropeltes* at the Museum of the Institute of Tropical Aquaculture, Universiti Malaysia Terengganu, Kuala Terengganu, Malaysia (UMTCM1 to UMTCM5)
