# Supplementary material for: Unraveling a 146 Years Old Taxonomic Puzzle: Validation of Malabar Snakehead, Species-Status and Its Relevance for Channid Systematics and Evolution
Source: PLoS One. 2011 Jun 24;6(6):e21272. doi: 10.1371/journal.pone.0021272 (PMC3123301; doi:10.1371/journal.pone.0021272)
Supplement: Table S2 — List of fish species used for the study, their NCBI accession numbers, voucher numbers and the respective museums. (PDF) [file pone.0021272.s005.pdf]

Table S2: List of fish species used for the study, their NCBI accession numbers, Voucher Numbers and the respective museums

Species	NCBI-Accession Number	Gene region (partial)	Voucher Number	Museum	Remarks
Channa diplogramma	EU342210	COI	CD6	FGL-FMNC	Specimen H in figure 4
Channa diplogramma	EU342209	COI	CD5	FGL-FMNC	Specimen G in figure 4
Channa diplogramma	EU342208	COI	CD4	FGL-FMNC	Specimen E in figure 4
Channa diplogramma	EU342207	COI	CD3	FGL-FMNC	Specimen D in figure 4
Channa diplogramma	EU342206	COI	CD2	FGL-FMNC	Specimen C in figure 4
Channa diplogramma	EU342205	COI	CD1	FGL-FMNC	Specimen A in figure 4
Channa striata	EU342204	COI	CS2	FGL-FMNC	
Channa striata	EU342203	COI	CS1	FGL-FMNC	
Channa punctata	EU342202	COI	CP2	FGL-FMNC	
Channa punctata	EU342201	COI	CP1	FGL-FMNC	
Channa marulia	EU342200	COI	CM2	FGL-FMNC	
Channa marulia	EU342199	COI	CM1	FGL-FMNC	
Channa gachua	EU342198	COI	CG2	FGL-FMNC	
Channa gachua	EU342197	COI	CG1	FGL-FMNC	
Channa bleheri	EU342196	COI	CB2	FGL-FMNC	
Channa bleheri	EU342195	COI	CB1	FGL-FMNC	
Channa aurantimaculata	EU342194	COI	CA2	FGL-FMNC	
Channa aurantimaculata	EU342193	COI	CA1	FGL-FMNC	
Channa diplogramma	EU342192	16S	CD6	FGL-FMNC	Specimen H in figure 4
Channa diplogramma	EU342191	16S	CD5	FGL-FMNC	Specimen G in figure 4
Channa diplogramma	EU342190	16S	CD4	FGL-FMNC	Specimen E in figure 4
Channa diplogramma	EU342189	16S	CD3	FGL-FMNC	Specimen D in figure 4
Channa diplogramma	EU342188	16S	CD2	FGL-FMNC	Specimen C in figure 4
Channa diplogramma	EU342187	16S	CD1	FGL-FMNC	Specimen A in figure 4
Channa striata	EU342186	16S	CS2	FGL-FMNC	
Channa striata	EU342185	16S	CS1	FGL-FMNC	
Channa punctata	EU342184	16S	CP2	FGL-FMNC	
Channa punctata	EU342183	16S	CP1	FGL-FMNC	
Channa marulia	EU342182	16S	CM2	FGL-FMNC	
Channa marulia	EU342181	16S	CM1	FGL-FMNC	
Channa gachua	EU342180	16S	CG2	FGL-FMNC	
Channa gachua	EU342179	16S	CG1	FGL-FMNC	
Channa bleheri	EU342178	16S	CB2	FGL-FMNC	
Channa bleheri	EU342177	16S	CB1	FGL-FMNC	
Channa aurantimaculata	EU342176	16S	CA2	FGL-FMNC	
Channa aurantimaculata	EU342175	16S	CA1	FGL-FMNC	
Channa micropeltes	JF900369	COI	UMTCM5	UMT	
Channa micropeltes	JF900370	16S	UMTCM5	UMT	

UMT = Institute of Tropical Aquaculture, Universiti Malaysia Terengganu, Kuala Terengganu, Malaysia , **FGL-FMNC** = Fish Genetics Lab – Fathima Matha National College, Kollam , Kollam, Kerala, India.
