# Supplementary material for: Unraveling a 146 Years Old Taxonomic Puzzle: Validation of Malabar Snakehead, Species-Status and Its Relevance for Channid Systematics and Evolution
Source: PLoS One. 2011 Jun 24;6(6):e21272. doi: 10.1371/journal.pone.0021272 (PMC3123301; doi:10.1371/journal.pone.0021272)
Supplement: Table S3 — Genetic distance values calculated for the partial mitochondrial COI sequences of different Channa species used in the study. (PDF) [file pone.0021272.s006.pdf]

Table S3: Genetic distance values calculated for the partial mitochondrial COI sequences of different *Channa* species used in the study

	CA1	CA2	CB1	CB2	CG1	CG2	CM1	CM2	CP1	CP2	CS1	CS2	Cmi4	CD1	CD2	CD3	CD4	CD5	CD6	NN
CA1	0,000																			
CA2	0,002	0,000																		
CB1	0,304	0,314	0,000																	
CB2	0,304	0,314	0,000	0,000																
CG1	0,327	0,337	0,397	0,397	0,000															
CG2	0,327	0,337	0,397	0,397	0,000	0,000														
CM1	0,533	0,548	0,662	0,662	0,576	0,576	0,000													
CM2	0,533	0,548	0,662	0,662	0,576	0,576	0,000	0,000												
CP1	0,507	0,521	0,538	0,538	0,554	0,554	0,403	0,403	0,000											
CP2	0,490	0,504	0,538	0,538	0,554	0,554	0,403	0,403	0,004	0,000										
CS1	0,699	0,717	0,849	0,849	0,633	0,633	0,512	0,512	0,457	0,473	0,000									
CS2	0,710	0,728	0,862	0,862	0,643	0,643	0,504	0,504	0,465	0,480	0,002	0,000								
Cmi4	0,673	0,690	0,762	0,762	0,809	0,809	0,554	0,554	0,466	0,466	0,662	0,653	0,000							
CD1	0,594	0,609	0,799	0,799	0,643	0,643	0,446	0,446	0,451	0,451	0,569	0,560	**0,210**	0,000						
CD2	0,594	0,609	0,799	0,799	0,643	0,643	0,446	0,446	0,451	0,451	0,569	0,560	**0,210**	0,000	0,000					
CD3	0,594	0,609	0,799	0,799	0,643	0,643	0,446	0,446	0,451	0,451	0,569	0,560	**0,210**	0,000	0,000	0,000				
CD4	0,555	0,570	0,749	0,749	0,601	0,601	0,415	0,415	0,438	0,438	0,562	0,554	**0,187**	0,007	0,007	0,007	0,000			
CD5	0,555	0,570	0,749	0,749	0,601	0,601	0,415	0,415	0,438	0,438	0,562	0,554	**0,187**	0,007	0,007	0,007	0,000	0,000		
CD6	0,555	0,570	0,749	0,749	0,601	0,601	0,415	0,415	0,438	0,438	0,562	0,554	**0,187**	0,007	0,007	0,007	0,000	0,000	0,000	
NN	1,341	1,373	1,234	1,234	1,101	1,101	0,878	0,878	0,959	0,972	0,939	0,939	0,983	0,928	0,928	0,928	0,871	0,871	0,871	0,000

CA = Channa aurantimaculata, CB = Channa bleheri, CG = Channa gachua, CM = Channa marulia,
CP = Channa punctata, CS = Channa Striata, Cmi = Channa micropeltes, CD = Channa diplogramma,
NN = Notopterus notopterus
