# Supplementary material for: Unraveling a 146 Years Old Taxonomic Puzzle: Validation of Malabar Snakehead, Species-Status and Its Relevance for Channid Systematics and Evolution
Source: PLoS One. 2011 Jun 24;6(6):e21272. doi: 10.1371/journal.pone.0021272 (PMC3123301; doi:10.1371/journal.pone.0021272)
Supplement: Table S4 — Genetic distance values calculated for the partial 16s rRNA gene sequences of different Channa species used in the study. (PDF) [file pone.0021272.s007.pdf]

| **Table S4:** Genetic distance values calculated for the partial 16s rRNA gene sequences of different *Channa* species used in the study | | | | | | | | | | | | | | | | | | | | | | | |
|---|---|---|---|---|---|---|---|---|---|---|---|---|---|---|---|---|---|---|---|---|---|---|---|
| | CA1 | CA2 | CB1 | CB2 | CG1 | CG2 | CM1 | CM2 | CP1 | CP2 | CS1 | CS2 | Cmi | Cmi4 | CD6 | CD5 | CD4 | CD3 | CD2 | CD1 | PCO | NN | CMNE |
| CA1 | 0.000 | | | | | | | | | | | | | | | | | | | | | | |
| CA2 | 0.002 | 0.000 | | | | | | | | | | | | | | | | | | | | | |
| CB1 | 0.040 | 0.044 | 0.000 | | | | | | | | | | | | | | | | | | | | |
| CB2 | 0.037 | 0.040 | 0.002 | 0.000 | | | | | | | | | | | | | | | | | | | |
| CG1 | 0.033 | 0.036 | 0.040 | 0.037 | 0.000 | | | | | | | | | | | | | | | | | | |
| CG2 | 0.031 | 0.034 | 0.042 | 0.039 | 0.002 | 0.000 | | | | | | | | | | | | | | | | | |
| CM1 | 0.132 | 0.127 | 0.162 | 0.156 | 0.137 | 0.133 | 0.000 | | | | | | | | | | | | | | | | |
| CM2 | 0.137 | 0.131 | 0.162 | 0.156 | 0.137 | 0.133 | 0.002 | 0.000 | | | | | | | | | | | | | | | |
| CP1 | 0.171 | 0.165 | 0.205 | 0.197 | 0.155 | 0.159 | 0.185 | 0.190 | 0.000 | | | | | | | | | | | | | | |
| CP2 | 0.171 | 0.165 | 0.205 | 0.197 | 0.155 | 0.159 | 0.185 | 0.190 | 0.000 | 0.000 | | | | | | | | | | | | | |
| CS1 | 0.140 | 0.134 | 0.144 | 0.138 | 0.138 | 0.134 | 0.089 | 0.089 | 0.214 | 0.214 | 0.000 | | | | | | | | | | | | |
| CS2 | 0.141 | 0.135 | 0.145 | 0.139 | 0.139 | 0.135 | 0.089 | 0.089 | 0.202 | 0.202 | 0.004 | 0.000 | | | | | | | | | | | |
| Cmi | 0.154 | 0.148 | 0.173 | 0.167 | 0.155 | 0.151 | 0.078 | 0.082 | 0.183 | 0.183 | 0.083 | 0.083 | 0.000 | | | | | | | | | | |
| Cmi4 | 0.161 | 0.154 | 0.180 | 0.173 | 0.161 | 0.157 | 0.082 | 0.086 | 0.190 | 0.190 | 0.087 | 0.088 | 0.002 | 0.000 | | | | | | | | | |
| CD6 | 0.169 | 0.163 | 0.180 | 0.174 | 0.172 | 0.167 | 0.088 | 0.092 | 0.214 | 0.214 | 0.087 | 0.088 | **0.024** | **0.027** | 0.000 | | | | | | | | |
| CD5 | 0.169 | 0.163 | 0.180 | 0.174 | 0.172 | 0.167 | 0.088 | 0.092 | 0.214 | 0.214 | 0.087 | 0.088 | **0.024** | **0.027** | 0.000 | 0.000 | | | | | | | |
| CD4 | 0.169 | 0.163 | 0.180 | 0.174 | 0.172 | 0.167 | 0.088 | 0.092 | 0.214 | 0.214 | 0.087 | 0.088 | **0.024** | **0.027** | 0.000 | 0.000 | 0.000 | | | | | | |
| CD3 | 0.169 | 0.163 | 0.180 | 0.174 | 0.172 | 0.167 | 0.088 | 0.092 | 0.214 | 0.214 | 0.087 | 0.088 | **0.024** | **0.027** | 0.000 | 0.000 | 0.000 | 0.000 | | | | | |
| CD2 | 0.169 | 0.163 | 0.180 | 0.174 | 0.172 | 0.167 | 0.088 | 0.092 | 0.214 | 0.214 | 0.087 | 0.088 | **0.024** | **0.027** | 0.000 | 0.000 | 0.000 | 0.000 | 0.000 | | | | |
| CD1 | 0.176 | 0.169 | 0.187 | 0.180 | 0.178 | 0.174 | 0.092 | 0.096 | 0.222 | 0.222 | 0.092 | 0.093 | **0.027** | **0.030** | 0.002 | 0.002 | 0.002 | 0.002 | 0.002 | 0.000 | | | |
| PCO | 0.135 | 0.130 | 0.181 | 0.174 | 0.155 | 0.151 | 0.152 | 0.157 | 0.194 | 0.194 | 0.176 | 0.177 | 0.152 | 0.158 | 0.179 | 0.179 | 0.179 | 0.179 | 0.179 | 0.186 | 0.000 | | |
| NN | 0.673 | 0.654 | 0.779 | 0.757 | 0.692 | 0.682 | 0.644 | 0.655 | 0.688 | 0.688 | 0.666 | 0.663 | 0.708 | 0.716 | 0.694 | 0.694 | 0.694 | 0.694 | 0.694 | 0.714 | 0.620 | 0.000 | |
| CMNE | 0.138 | 0.133 | 0.164 | 0.158 | 0.139 | 0.135 | 0.021 | 0.023 | 0.194 | 0.194 | 0.080 | 0.080 | 0.077 | 0.081 | 0.087 | 0.087 | 0.087 | 0.087 | 0.087 | 0.091 | 0.150 | 0.600 | 0.000 |

*CA = Channa aurantimaculata. CB = Channa bleheri. CG = Channa gachua. CM = Channa marulia.*

*CP = Channa punctata. CS = Channa Striata. Cmi = Channa micropeltes. CD = Channa diplogramma.*

*PCO = Parachanna obscura. NN = Notopterus notopterus. CMNE = Channa marulia from North East India*
